# Depression, Drinking to Cope, and Alcohol Use Severity Among Latinos Who Drink: The Moderating Role of Sex

**DOI:** 10.1002/jclp.23755

**Published:** 2024-11-29

**Authors:** Cory L. Cobb

**Affiliations:** ^1^ Department of Health Behavior, School of Public Health Texas A&M University College Station Texas USA

**Keywords:** alcohol use, coping, depressive symptoms, gender, Latinos, sex

## Abstract

**Objectives:**

The present study evaluated sex differences in the direct and indirect links between depressive symptoms, coping motives to drink, and alcohol use severity among Latinos who drink.

**Methods:**

A large and diverse panel sample of Latinos, who were strategically sampled to be reflective of the Latino demographics of the state of Texas, completed questionnaires assessing their depressive symptomatology, coping motives to drink, and alcohol use behaviors.

**Results:**

Direct effects were significant in hypothesized directions such that depressive symptoms was positively associated with both coping motives to drink and alcohol use severity, and drinking motives to cope were positively associated with alcohol use severity. Moderator analysis showed that, compared to Latina women, the association of depressive symptoms with both drinking to cope and alcohol use severity was significantly higher among Latino men. Regarding indirect effects, a moderated mediation analysis showed that the indirect effect of depressive symptoms on alcohol use severity through coping motives to drink was statistically significant for both Latino/a men and women; however, the index of moderated mediation showed that this indirect effect was significantly greater among Latino men.

**Conclusion:**

Although both Latino/a men and women may consume alcohol and turn to alcohol to drink with negative affect associated with depressive symptoms, this process may be stronger among Latino men. Findings are discussed considering prior work and implications for practitioners.

## 1 Introduction

Latinos in the United States occupy a disadvantaged social position that increases their risk for experiencing depressive symptomatology (Cobb et al. 2021; Stryker et al. 2021). Research indicates that the depressive symptoms are frequently associated with higher alcohol consumption, and Latinos who drink experience more adverse consequences of alcohol misuse compared to non‐Latino Whites (Cano et al. 2017; Jetelina et al. 2016; Mulia et al. 2009). Yet, alcohol misuse among Latinos remains understudied relative to other populations (i.e., Whites), and the mechanisms linking depressive symptoms to alcohol misuse among Latinos—including whether the effects of these mechanisms vary as a function of biological sex—are largely unknown. Following motivational models of alcohol use (Cooper et al. 2016), coping motives—drinking to avoid, escape, or regulate negative emotions—may help to explain the link between depressive symptoms and alcohol misuse among Latinos who drink. Further, the extent to which Latinos drink alcohol, including the degree to which they drink to cope, likely varies between Latino/a men and women due to differential alcohol use socialization (Cano et al. 2017, 2023).

The purpose of the present study is to (1) evaluate whether depressive symptoms is linked to greater alcohol use severity indirectly through coping motives to drink among a large and diverse sample of Latinos who drink, and (2) assess whether the indirect effect of coping motives in the relationship between depressive symptoms and alcohol misuse varies between men and women. The present study will inform the clinical literature by elucidating a possible mechanism linking depressive symptoms to alcohol use among an understudied population and by determining whether these links vary between Latino/a men and women.

### 1.1 Depression, Drinking to Cope, and Alcohol Use Severity Among Latinos

Alcohol is among the most misused substances in the United States and is a leading cause of preventable death (Centers for Disease Control and Prevention 2024). Studies on alcohol misuse among Latinos who drink are needed as research indicates that, although Latinos may drink less on average compared to non‐Latino Whites, particularly Latino immigrants, they often experience more severe consequences associated with alcohol misuse (e.g., Salas‐Wright et al. 2018; Villalobos and Bridges 2018). For example, compared to non‐Latino Whites, research indicates that Latinos who choose to drink often consume alcohol in higher volumes and experience more adverse alcohol‐related problems (e.g., job loss, legal citations, and liver disease; Mulia et al. 2009; Vaeth, Wang‐Schweig, and Caetano 2017). Thus, the public health impact of alcohol misuse among Latinos who drink is significant, and research is needed that identifies proximal correlates of alcohol use (drinking to cope) to offset risk for alcohol misuse and related problems.

Research has consistently linked the experience of depressive symptoms to higher alcohol consumption (e.g., Cano et al. 2017; Cobb et al. 2021; Jetelina et al. 2016). Although there is evidence that the relationship between depressive symptoms and alcohol use is likely bidirectional (Brière et al. 2014; Pacek, Martins, and Crum 2013), according to motivational models of alcohol use (Cooper et al. 2016), there are multiple motives for drinking alcohol (e.g., social, conformity, enhancement). However, individuals experiencing depressive symptoms may be more likely to drink alcohol to cope (coping motives) with the negative feelings and thoughts that are associated with depression (Hogarth et al. 2018). Longitudinal studies across both youth and adult populations have found that drinking alcohol to cope with depressive symptoms was associated with greater risk of experiencing alcohol‐related problems, and higher levels of depressive symptoms were related to subsequent alcohol misuse (e.g., Collins et al. 2018; Magee and Connell 2021). Given the links between depressive symptoms, coping motives to drink, and alcohol use, it is expected that greater levels of depressive symptoms will be positively associated with higher alcohol consumption indirectly through drinking to cope.

### 1.2 Sex as a Moderator

Although some studies have considered the potential mechanisms linking depressive symptoms to alcohol use, there is a dearth of research of these relationships among underserved Latino populations and even less research considering whether these links vary across salient social categories (i.e., sex). Studies have found significant sex differences in alcohol use severity among Latinos who drink such that Latino men tend to drink more heavily than Latina women, and Latina women may be less prone than Latino men to turn to alcohol as a coping mechanism for experiencing depressive symptoms (Castañeda et al. 2019; Rote and Brown 2013).

Despite myriad explanations for differential alcohol use behaviors between men and women, one of the most common explanations for this pattern is that men are more likely to turn to alcohol to cope and drink more heavily due to traditional gender norms around alcohol use (Perrotte and Zamboanga 2021). That is, men are often more socialized to think of alcohol use as an indicator of their masculinity, whereas women may refrain from heavier drinking to avoid appearing masculine or being criticized (Erol and Karpyak 2015; Huselid and Cooper 1992). Differential gender socialization around alcohol use is also prevalent among Latino populations—especially as it relates to the traditional masculine norm of *machismo* in which frequent alcohol use represents typical masculine behavior within the Latino population (Arciniega et al. 2008; Perrotte and Zamboanga 2021; Perrotte, Zamboanga, and Kearns 2020).

Given decades of research on sex differences and gender socialization in alcohol use and coping behaviors, it is expected that sex should moderate the indirect effects of drinking alcohol to cope in the relationship between depressive symptomatology and alcohol use severity. That is, men should report both greater levels of alcohol use severity as well as coping motives to drink.

### 1.3 The Present Study

**Figure 1:**
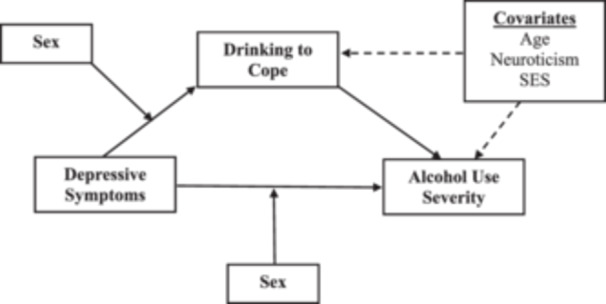
Conditional process model of the links between depressive symptoms, drinking to cope, alcohol use severity, and sex.

The present study will evaluate the direct and indirect links between depressive symptoms, coping motives to drink, and alcohol use severity among a large and diverse sample of Latinos who drink across Texas. In addition, sex will be assessed as a potential moderator to determine whether these links—including both direct and indirect effects—vary in direction and/or magnitude between Latino/a men and women. Grounded in the motivational theory of alcohol use (Cooper et al. 2016), it is hypothesized that (a) depressive symptoms will be positively associated with both coping motives to drink and alcohol use severity; (b) depressive symptoms will be associated with alcohol use severity indirectly through coping motives to drink; and (c) sex will moderate these links such that, compared to Latina women, Latino men who drink will report higher levels of drinking to cope and alcohol use severity. Accordingly, the indirect link between depressive symptoms and alcohol use severity through drinking to cope should be larger among Latino men than among Latina women (Figure 1).

## 2 Materials and Methods

### 2.1 Sample and Procedures

Data for the present study were collected using a professional data collection research firm (ReconMR) located in Central Texas with additional field offices throughout the state. Data were collected from October 2023 to December 2023, and the study was finalized in January 2024. Using VOXCO software, the research firm distributed the study survey provided by the research team to a recruited panel sample of 800 Latino adults between the ages of 18–65 across the state of Texas. According to recent demographic estimates (Texas Demographic Center 2024), approximately 83% of Latinos in Texas are of Mexican heritage, 17% are of Central and South American heritage, and 72% are US‐born individuals. To obtain a sample as representative as possible of the Latino population in Texas, the research firm set recruitment quotas to align with these population demographic estimates.

Moreover, to further increase generalizability, heterogeneity quotas were set to recruit an equal number of Latino/a men and women as well as to recruit Latinos from both metropolitan (Dallas, Fort Worth, Austin, El Paso, San Antonio, Houston) and nonmetropolitan areas across Texas. The final sample aligned closely with Texas demographics such that 84% were of Mexican heritage, and the remaining 16% were of other Hispanic or Latino heritages. Regarding sex and location, the final sample consisted of 400 men and 400 women, and 313 resided in metropolitan areas. However, the final sample consisted of 11% immigrants versus the 28% quota target that was set by the research team. To align the sample distribution with Texas population demographics, we provided statistical frequency weights to the data based on immigrant status (Pfeffermann 1993). Thus, the final sample was relatively reflective of the Latino population in Texas. Once the sample was recruited, ReconMR sent the study surveys to participants who provided informed consent before completing the surveys. Although participants were provided a response option to not respond to a demographic question, they were not allowed to skip questions during the survey. For this reason, there are no missing data for survey items. Participants received monetary compensation at a rate they agreed upon with ReconMR before participation. The study was approved by the Institutional Review Board of the first author.

During administration of the original survey, participants were asked whether they currently consume alcohol (to any degree). Because the present study centers on alcohol misuse, the 547 participants who reported consuming alcohol were included in the analysis—those that reported no alcohol consumption (*n* = 253) were excluded from data analysis. The average age was 35 years (SD = 10.97, median = 34, range = 18–65). Regarding biological sex assigned at birth, 293 identified as male, and 254 identified as female. No individuals identified as any gender outside of male or female. For nativity status, 490 reported being born in the United Sates whereas 57 reported being born in 14 other Latin American countries. Regarding location, 220 reported living in metropolitan areas, and 326 reported living outside of metropolitan areas (1 participant did not respond). Participants were asked the amount of money they and their family lived on last month after taxes (including public assistance): 36.9% reported living $2000 or more, 24.3% on $1000–$1999, 15.2% on $500–$999, 9.1% on $200–$499, and 14.4% on $200 or less. Regarding education, participants reported having a college degree (30.5%), attending some college (32.7%), holding a high school diploma (26%), completed a GED (5.1%), and never finished high school (5.6%). Finally, 96% completed the surveys in English, whereas the remaining 4% completed surveys in Spanish.

### 2.2 Measures

#### 2.2.1 Depressive Symptoms

The Patient Health Questionnaire‐9 (PHQ‐9; Kroenke, Spitzer, and Williams 2001) was used to measure depressive symptoms. The PHQ‐9 is a well‐established, self‐reported index of depressive symptoms with excellent psychometric properties (e.g., Beard et al. 2016; Kroenke, Spitzer, and Williams 2001). The PHQ‐9 consists of nine items that are scored on a Likert‐type scale, ranging from 0 (*not at all*) to 3 (*nearly every day*), and asks participants to report on depressive symptoms over the last 2 weeks (e.g., feeling down or hopeless, little interest or pleasure in doing things). Higher scores reflect greater levels of depressive symptoms. The PHQ‐9 has a sensitivity of 88% and specificity of 88% for detecting major depressive disorders at a clinical cutoff of 10 (Kroenke, Spitzer, and Williams 2001). The Spanish version of the PHQ‐9 was provided those who preferred to complete the measure in Spanish (Muñoz‐Navarro et al. 2017). Cronbach's *α* was 0.94. Note that the English and Spanish versions were included together in all reliability analyzes given the small number of individuals who completed the measures in Spanish.

#### 2.2.2 Drinking to Cope

The Coping subscale of the well‐established Drinking Motives Questionnaire‐Revised (DMQ‐R; Cooper 1994) was used to measure coping motives to drink alcohol. The Coping subscale consists of five self‐reported items that reflect coping motives for alcohol use. Items are scored on a Likert‐type scale ranging from 1 (*almost never/never*) to 5 (*almost always/always*) with higher scores reflecting higher levels of drinking to cope. Example items include reporting frequency of drinking “To forget your worries” or “Because it helps you when you feel depressed or nervous.” For Spanish‐speaking respondents, a validated Spanish version of the DMQ‐R was used (Mezquita et al. 2016). Internal consistency was strong at 0.93.

#### 2.2.3 Alcohol‐Use Severity

The Alcohol Use Disorder Identification Test (AUDIT) was used to measure alcohol use severity. The AUDIT was originally developed by the World health Organization to identify problematic drinking behaviors in primary care settings (Babor et al. 2001). The AUDIT consists of 10 self‐reported items that range from 0 (*never*) to 4 (*four or more times per week*). Example items include “How often do you have a drink containing alcohol” and “How often do you have six or more drinks on one occasion.” Higher scores reflect greater alcohol use severity. For those who completed the measure in Spanish, a validated Spanish version of AUDIT was used (Rubio Valladolid et al. 1998). Cronbach's *α* in the present study was strong at 0.93, indicating good internal consistency.

#### 2.2.4 Sex

Sex as a moderating variable was measured by asking participants to report on the biological sex they were assigned at birth. Responses were coded as 0 for female and 1 for male. The study survey also asked participants about intersex, but no respondents identified as intersex.

#### 2.2.5 Covariates

The covariates of age, socioeconomic status, and neuroticism (negative emotionality) will be statistically controlled given their consistent links with depressive symptoms, coping behaviors, and alcohol misuse (e.g., Castañeda et al. 2019; Chinneck et al. 2018; Fischer et al. 2007). For covariates, we measured age in years, socioeconomic status (monthly household income after taxes), and neuroticism (e.g., Castañeda et al. 2019; Chinneck et al. 2018; Fischer et al. 2007). Neuroticism is a measure of negative emotionality and was measured using the neuroticism subscale of the well‐established Big Five Inventory (John, Donahue, and Kentle 1991). The subscale consists of eight items that asks participants to rate the extent to which they agree with certain personality features that correspond to a neurotic personality trait (e.g., tends to worry a lot, is often moody, doesn't' remain calm in tense situations). For primarily Spanish‐speaking individuals, we used the validated Spanish version of the subscale (Benet‐Martínez and John 1998). Items are scored on a Likert‐type scale ranging from 1 (*disagree strongly*) to 5 (*agree strongly*), and three positively‐worded items are reverse coded. Higher scores reflect greater negative emotionality. Cronbach's *α* for neuroticism was adequate at 0.76.

### 2.3 Analytical Plan

Analyzes proceeded in two steps. First, core assumptions of linearity, multicollinearity, and outliers that underlie the analytical model were assessed. After checking statistical assumptions, descriptive statistics were computed as preliminary analyzes. Second, to test the moderated mediation model (Figure 1), we employed nonparametric bootstrapping using ordinary least squares regression. This approach provides a more powerful test of indirect effects than normal theory approaches and more closely approximates the sampling distribution of indirect effects. Bootstrapping also represents a robust method of testing indirect effects while controlling for Type‐I errors (Preacher and Hayes 2008). Furthermore, PROCESS software was used with 95% confidence intervals (CIs) corrected for bias across 10,000 bootstrapped resamplings to test the indirect effects of drinking to cope in the relationship between depressive symptoms and alcohol use severity, as well as the moderating role of sex on these effects (Hayes 2018). Variables were mean‐centered before testing interaction effects. If a *CI* does not contain zero, then a significant indirect effect may be inferred. An index of moderated mediation is provided that indicates whether the indirect effects of drinking to cope are significantly different between Latino/a men and women (Hayes 2018).

## 3 Results

### 3.1 Preliminary Results

Before testing the theoretical model, assumptions of linearity, multicollinearity, and outliers were assessed. Bivariate scatter plots of predictors and outcomes showed no violations of linearity. Examination of the correlation matrix showed no evidence of multicollinearity (Table 1). The influence of outliers were examined by computing Mahalanobis distances followed by comparing obtained values to critical values of the *χ* ^2^ distribution (Ghorbani 2019). None of the obtained values exceeded the critical values, indicating no significant outlier effects.

**Table 1:** Bivariate correlations of main study variables and covariates.

Variable	1	2	3	4	5	6	7
1. Depression	—						
2. Drinking to cope	0.50**	—					
3. Alcohol‐use severity	0.53**	0.64**	—				
4. Neuroticism	0.50**	0.33**	0.22**	—			
5. Age	−0.16**	−0.14*	−0.12*	−0.26**	—		
6. SES	−0.04	−0.02	0.025	−0.06	0.08	—	
7. Sex	0.11*	0.25**	0.32**	−0.13*	0.10*	0.08	—
*M*	10.41	12.93	11.35	24.53	35.23		
SD	8.00	6.38	10.27	6.12	10.97		
Skew	0.31	0.36	0.88	−0.47	0.62		

*Note:* Correlations of continuous variables with sex represent point‐biserial correlations.

Abbreviations: M = mean, SD = standard deviation.

** < 0.001,

* < 0.01.

Descriptive analyzes showed that there were considerable experiences of depressive symptoms as 49% of participants reported mean level symptoms at or above the clinical cutoff of 10 (Kroenke et al. 2010), with more men (*n* = 160) reporting mean level symptoms above the cutoff than women (*n* = 108). Similarly, approximately 23% of drinkers reported an overall score at or above the clinical cutoff of 20, indicating significant alcohol use and/or possible dependence in the sample (Higgins‐Biddle and Babor 2018). Men (*n* = 99) were more represented among those exceeding the cutoff compared to women (*n* = 31).

### 3.2 Primary Results

#### 3.2.1 Direct Effects

Depressive symptoms were positively associated with drinking to cope (*b* = 0.24, *p* < 0.0001, CI_95%_ = 0.15–0.33) and to alcohol use severity (*b* = 0.23, *p* < 0.001, CI_95%_ = 0.11–0.36). Drinking to cope was positively associated with alcohol use severity (*b* = 0.71, *p* < 0.0001, CI_95%_ = 0.60–0.83). For sex, compared to Latina women, Latino men reported significantly higher coping motives to drink (*b* = 3.00, *p* < 0.0001, CI_95%_ = 2.07–3.92) and alcohol use severity (*b* = 3.57, *p* < 0.0001, CI_95%_ = 2.28–4.87). Among covariates, only neuroticism was significantly and positively associated with drinking to cope (*b* = 0.15, *p* < 0.001, CI_95%_ = 0.06–0.23) and alcohol use severity (*b* = −0.13, *p* < 0.05, CI_95%_ = −0.25–−0.01).

#### 3.2.2 Moderating Effects

**Figure 2:**
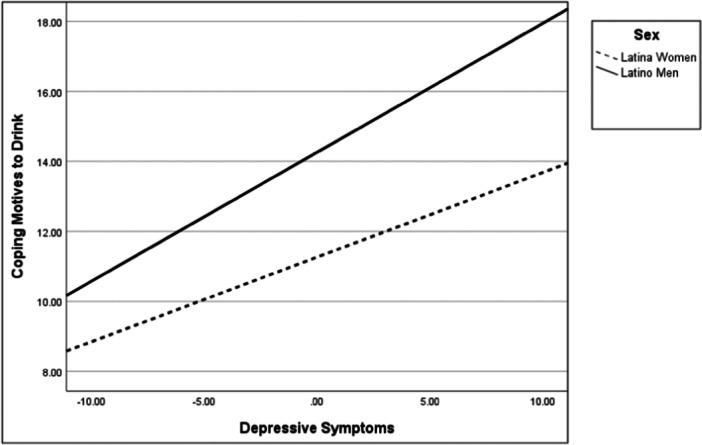
Moderating role of sex on depressive symptoms and coping motives to drink.

**Figure 3:**
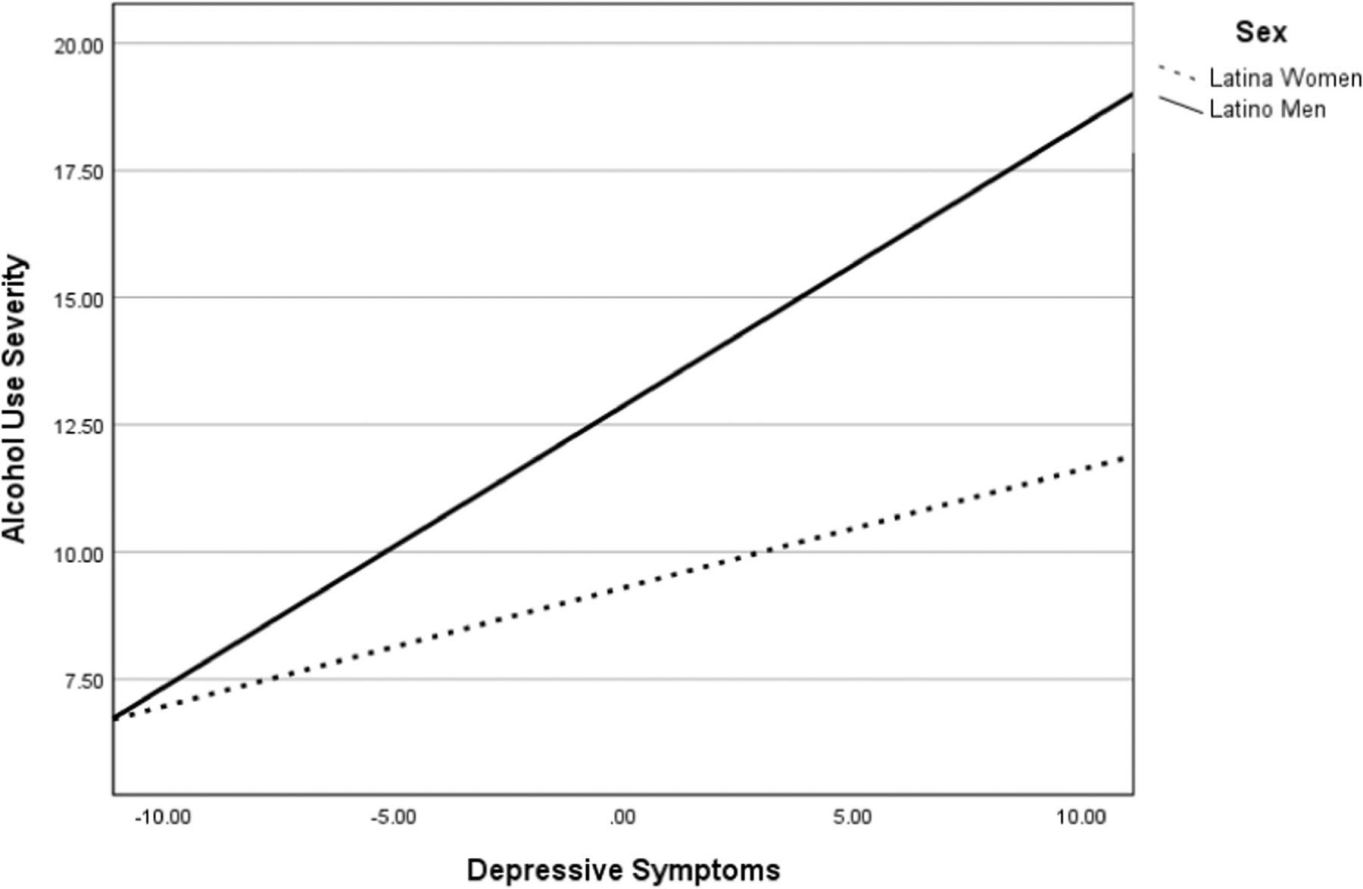
Moderating role of sex on depressive symptoms and alcohol use severity.

Following our model (Figure 1), results of the interactions showed that sex moderated the relationships between depressive symptoms and drinking to cope (*b* = 0.13, *p *< 0.05, CI_95%_ = 0.01–0.24) as well as with alcohol use severity (*b* = 0.32, *p* < 0.001, CI_95%_ = 0.17–0.47). Figures 2 and 3 show that the relationships between depressive symptoms and both drinking to cope and alcohol‐use severity were significantly stronger for men than for women.

#### 3.2.3 Conditional Indirect Effects

Because the theoretical model tested (Figure 1) is a moderated mediation model, the indirect effect of drinking to cope is conditioned on sex (Hayes 2018). Therefore, the indirect effect of depressive symptoms on alcohol use through drinking to cope is presented separately for men and women. Results showed that the indirect effect of depressive symptoms to alcohol use severity through coping motives to drink was significant for both Latino men (*b* = 0.26, CI_95%_ = 0.19–0.34) and Latina women (*b* = 0.17, CI_95%_ = 0.10–0.25). The index of moderated mediation was then computed to assess whether the difference in magnitude of the indirect effects between men and women were statistically significant. Results showed a statistically significant index of moderated mediation (Index = 0.09, CI_95%_ = 0.01–0.18). These results suggest that the indirect effect of depressive symptoms on alcohol use through drinking to cope was significantly stronger for Latino men than for Latina women.

## 4 Discussion

The present study examined the direct and indirect links between depressive symptoms, coping motives to drink, and alcohol‐use severity—including whether these associations varied across sex—among a large and diverse sample of Latinos. Results showed that depressive symptoms were positively associated with both coping motives to drink and alcohol use severity, and coping motives to drink were positively associated with alcohol use severity. Moreover, mediation analysis indicated that depressive symptoms were positively associated with alcohol use severity indirectly through coping motives to drink for both Latino/a men and women. However, the magnitude of the indirect effect of coping motives to drink was significantly stronger for Latino men than for Latina women.

First, and in line with the present study's hypotheses, depressive symptoms were positively associated with both greater coping motives to drink and higher alcohol consumption. This result aligns with prior literature that found positive links between depressive symptoms and alcohol use among Latinos (Cano et al. 2017; Cobb et al. 2021; Jetelina et al. 2016), as well as with research that found positive associations between depressive symptoms and drinking to cope. For example, using experimental methods, Hogarth et al. (2018) found that negative mood states (i.e., depressive symptoms) were associated with alcohol‐seeking behaviors and that individuals who reported depressive symptoms and/or drinking to cope were more sensitive to the effects of negative mood on alcohol use behaviors. In a separate experiment, Hogarth and Hardy (2018) found that individuals who drink to cope with negative affect were more sensitive to the influence of depressed mood on alcohol‐seeking behaviors. These findings suggest that, although depressive symptoms and alcohol use likely has a bidirectional relationship (Brière et al. 2014; Pacek, Martins, and Crum 2013), individuals experiencing depressive symptoms may be more likely to drink alcohol to cope with the negative feelings that are associated with depression.

Second, and extending the literature, the present study found that the links between depressive symptoms, drinking to cope, and alcohol use severity were moderated by sex among Latinos. Specifically, the magnitude of the effects of depressive symptoms on drinking to cope and alcohol use severity were greater among Latino men compared to Latina women. Although gender norms were not tested explicitly in the present study, research suggests that Latino men are more likely to turn to alcohol to cope and to drink more heavily due to traditional gender norms around alcohol use (Perrotte and Zamboanga 2021). Several studies indicate that men are more socialized to drink alcohol use as an indicator of masculinity, whereas women refrain from heavier drinking to avoid criticism or appearing masculine (Erol and Karpyak 2015; Huselid and Cooper 1992). Within Latino culture, the value of *machismo* may further increase alcohol use among men given that this value entails the notion that alcohol use is an indicator of masculinity (Arciniega et al. 2008; Perrotte and Zamboanga 2021; Perrotte, Zamboanga, and Kearns 2020).

Although the direct links between depressive symptoms, drinking to cope, and alcohol use severity have been considered in prior work, there is a dearth of research, especially among Latinos, on whether depressive symptoms may be associated with alcohol use indirectly through coping motives to drink—and no research has examined whether these direct and indirect links differ between Latino/a men and women. The present study found that the indirect effects of depressive symptoms on alcohol use through drinking to cope was significant for both men and women, thus providing evidence for coping motives to drink as a potential mediator of the depression‐alcohol use relationship. However, moderated mediation analysis revealed that the magnitude of the indirect effect was significantly higher for Latino men than for Latina women. This result suggests that, although both Latino/a men and women may consume alcohol and turn to alcohol to drink with negative affect, this process may be stronger among Latino men.

### 4.1 Implications

The present study has both theoretical and practical implications for clinical research and practice. First, it is among the first studies to assess sex differences in the direct and indirect links between depressive symptoms, coping motives to drink, and alcohol use severity among Latinos. Findings suggest that Latino/a men and women may differ in the degree to which they turn to alcohol to cope with the negative affect associated with depressive symptoms. Clinical practitioners working with Latinos experiencing depressive symptoms may consider that men may be more likely to turn to alcohol as a coping mechanism and may consume alcohol at higher rates than Latina women. Practitioners may also consider exploring cultural gender norms around alcohol use given prior research that consistently shows positive associations between traditional masculine gender norms and alcohol consumption within Latino culture. This may entail exploring cultural beliefs and gender norms around mental health and alcohol use behaviors, as well the ways in which such behaviors serve as coping mechanisms for depressive symptoms. Moreover, neuroticism emerged as a significant correlate of both drinking to cope as well as with alcohol use severity. Clinicians working with Latinos who drink may consider negative emotionality as a personality trait that makes their client smore susceptible to using alcohol to cope and thus to experience greater alcohol use severity. Finally, because alcohol use is associated with more alcohol‐related problems (e.g., aggression, job loss, legal citations, and liver disease) compared to non‐Latino Whites, practitioners may consider the myriad health risk factors that covary with depression and alcohol use among this vulnerable population (Mulia et al. 2009; Vaeth, Wang‐Schweig, and Caetano 2017).

### 4.2 Limitations and Conclusion

Findings from the present study should be interpreted considering several limitations. First, all data were cross‐sectional which inhibits inferences around directionality and causality of pathways. Future research should employ longitudinal methodologies that permit stronger inference around directionality of pathways. Second, data were based on self‐reported measures of mental and behavioral health, which rely on past recollection of events. Third, data were collected among Latinos in Texas, which may not generalize to other contexts. Fourth, there were many nationalities represented in the present study, but small numbers of participants in each cell did not allow for formal statistical testing of difference in national drinking behaviors. It is possible that differences in drinking norms between nationalities may influence alcohol use behaviors in the present sample. Future research may consider differences in drinking behaviors and norms between Latinos from differing nationalities, including the ways in which such nationalities vary according to their conceptions of mental health, cultural and gender norms around drinking behaviors, and alcohol‐related consequences. Future work may also identify protective behavioral strategies that may buffer against harmful alcohol use behaviors among Latinos who drink. Fifth, only the coping motives scale was used in the present study. The primary reason for this decision is that drinking to cope is the motive that is most strongly and consistently linked to depression and alcohol use severity. Future research may consider the ways in which other drinking motives operate among Latinos experiencing depressive symptoms and their alcohol use severity.

These limitations notwithstanding, the present study is among the first to evaluate a theory‐driven model of mental health, coping behavior, and alcohol use among a large and diverse sample of Latinos—it is also the first to assess sex differences in both the direct and indirect effects of these relationships among Latino populations. As the Latino population continues to grow in the United States, and considering the myriad stressors they face as the largest ethnic minority group in the country, it will be critical to advance our understanding of this vulnerable population vis‐à‐vis their mental health challenges and the ways in which they may turn to maladaptive coping behaviors to manage these challenges. The present study was designed to increase such understanding of this population with the hope that other researchers and practitioners will build upon this important line of work.

## Ethics Statement

The study was approved by the Texas A&M Institutional Review Board (IRB2023‐0855M).

## Conflicts of Interest

The author declares no conflicts of interest.

## Data Availability

The data used for all analyzes, along with data code and study protocol, can be found on the Open Science Framework at http://osf.io/kf4ve/.
